# bFGF attenuates endoplasmic reticulum stress and mitochondrial injury on myocardial ischaemia/reperfusion *via* activation of PI3K/Akt/ERK1/2 pathway

**DOI:** 10.1111/jcmm.12346

**Published:** 2014-12-23

**Authors:** Zhouguang Wang, Yue Wang, Junming Ye, Xianghong Lu, Yi Cheng, Lijun Xiang, Li Chen, Wenke Feng, Hongxue Shi, Xichong Yu, Li Lin, Hongyu Zhang, Jian Xiao, Xiaokun Li

**Affiliations:** a Department of Biochemistry and Molecular Biology, College of Basic Medical Science, Jilin UniversityChangchun, China; b School of Pharmacy, Key Laboratory of Biotechnology and Pharmaceutical Engineering, Wenzhou Medical UniversityWenzhou, Zhejiang, China; c Department of Anesthesia, The First Affiliated Hospital, Gannan Medical CollegeGanzhou, China; d Translation Medicine Research Center, Lishui People's Hospital, Wenzhou Medical UniversityLishui, China

**Keywords:** bFGF, endoplasmic reticulum stress, mitochondrial dysfunction, myocardial ischaemia/reperfusion

## Abstract

Extensive research focused on finding effective strategies to prevent or improve recovery from myocardial ischaemia/reperfusion (I/R) injury. Basic fibroblast growth factor (bFGF) has been shown to have therapeutic potential in some heart disorders, including ischaemic injury. In this study, we demonstrate that bFGF administration can inhibit the endoplasmic reticulum (ER) stress and mitochondrial dysfunction induced in the heart in a mouse model of I/R injury. *In vitro*, bFGF exerts a protective effect by inhibiting the ER stress response and mitochondrial dysfunction proteins that are induced by tert-Butyl hydroperoxide (TBHP) treatment. Both of these *in vivo* and *in vitro* effects are related to the activation of two downstream signalling pathways, PI3K/Akt and ERK1/2. Inhibition of these PI3K/Akt and ERK1/2 pathways by specific inhibitors, LY294002 and PD98059, partially reduces the protective effect of bFGF. Taken together, our results indicate that the cardioprotective role of bFGF involves the suppression of ER stress and mitochondrial dysfunction in ischaemic oxidative damage models and oxidative stress-induced H9C2 cell injury; furthermore, these effects underlie the activation of the PI3K/Akt and ERK1/2 signalling pathways.

## Introduction

Despite current optimal treatment, ischaemic heart disease is still the leading cause of death throughout the world 1. Because of the lack of effective therapies, myocardial ischaemia/reperfusion (I/R) injury remains a major medical problem today 2. The current standard treatment for myocardial ischaemia is rapid reperfusion, which can attenuate myocardial infarction, reduce cardiomyocyte apoptosis and restore contractile dysfunction. Reduction in infarct size by reperfusion in patients with acute myocardial infarction was also quickly translated to clinical practise 3; however, reperfusion also has the potential for additional injury, the overproduction of reactive oxygen species (ROS), mitochondrial dysfunction and overloading of calcium in the early reperfusion period. Unbalanced and high steady-state levels of reactive oxygen and nitrogen species (ROS/RNS) are responsible for cytotoxicity, which in turn leads to contractile dysfunction and cell death 4. Oxidative stress resulting from the overload of toxic ROS, such as hydroperoxide, also leads to various modifications of proteins, DNA, and lipids that induce cell proliferation, growth arrest, apoptosis or necrosis 5,6. Therefore, therapeutic strategies focusing on delaying or inhibiting apoptosis induced by oxidative stress may facilitate the treatment of myocardial I/R injury.

Basic fibroblast growth factor (bFGF or FGF-2) regulate a variety of biological functions including proliferation, morphogenesis and the suppression of apoptosis 7; it is an important angiogenic factor produced by hearts subjected to ischaemia. It acts on cells through transmembrane receptors with tyrosine kinase activity. Activation of FGFR induces a variety of intracellular signalling cascades, including the MAPK/ERK and PI3K/Akt pathways 8. In the heart, bFGF expression was shown to be up-regulated after cardiac injury, such as ischaemia/reperfusion, or in the process of cardiac remodelling 9. Moreover, the overexpression of bFGF increases cardiac myocyte viability after injury in isolated mouse hearts 10. bFGF delivered during reperfusion protects the heart against ischaemia-reperfusion injury through increased relative levels of PKC subtypes alpha, epsilon and zeta 11. In our previous study, we also proved that bFGF protects the heart against I/R-induced oxidative damage and cell death; however, the molecular mechanism by which bFGF treatment reduces myocardial I/R injury is unknown.

The increased generation of free radicals without a concomitant increase in antioxidant protection has been shown to induce apoptosis during I/R 12. The resulting oxidative stress leads to the peroxidation of phospholipid cardiolipin in the inner mitochondrial membrane, which contributes to the induction of mitochondrial fragmentation and dysfunction and triggers apoptosis 13,14. Mitochondrial processes have been demonstrated to be major events during apoptosis, and the Bcl-2 family, including Bax and Bak, is involved in the alteration of mitochondrial membrane potential as well as the release of mitochondrial apoptotic factors 15. Mitochondria are crucial signalling elements and potential effectors. The mitochondrial respiratory chain accepts electrons from NADH/H and flavine adenine dinucleotide (FADH)/H and transports them over four complexes ultimately onto oxygen generates, creating a proton gradient that then drives adenosine triphosphate (ATP) production 16. A previous study showed that exogenous taurine provides cardioprotection against myocardial ischaemic reperfusion by regulating the mitochondrial dysfunction induced by ROS generation 17. In addition, myocardial I/R injury is exacerbated by the promotion of myocardial mitochondrial dysfunction. The role and clinical impact of apoptosis needs to be further assessed to enable the development of more effective therapies to prevent myocardial damage during I/R. Such therapies must prevent mitochondrial dysfunction to preserve myocardial physiology. Although many reports have identified mitochondrial apoptosis as critical for myocardial I/R, whether bFGF may reduce myocardial mitochondrial dysfunction remains unclear.

Concurrently, endoplasmic reticulum (ER) stress is often accompanied by increased ROS generation in the myocardium 18,19. Excessive ROS production, which initiates the perturbation of the cellular redox balance, causes cell apoptosis 20,21. ROS generation appears to be one of the important stimuli that triggers ER stress 22, a paradigm called ‘ROS-dependent ER stress’. ER stress plays an important role in cell growth, differentiation and apoptosis. A previous study provided evidence for the involvement of ER stress in the cardiac apoptosis in the myocardial I/R model 23. These experimental data suggested that ER stress was initiated in myocardial I/R hearts, and the ER stress-induced apoptosis took part in the pathogenesis and development of myocardial I/R injury. Investigations have also demonstrated that ER stress induces cell apoptosis independently from mitochondria and death receptor-dependent pathways 24. New research also suggests that the ROS-stimulated activation of the PERK signalling pathway, rather than the IRE1 or activating transcription factor 6 (ATF-6) signalling pathways, is primarily responsible for ROS-mediated ER stress-induced myocyte apoptosis 18. Although many reports have identified that ER stress-induced apoptosis is critical for myocardial I/R, whether bFGF ameliorates myocardial ER stress is not clear.

In this study, we demonstrated that ER stress and mitochondrial dysfunction-induced apoptosis under oxidative stress *in vitro* and *in vivo*. bFGF inhibits ER stress and mitochondrial dysfunction-related protein expression *via* activation of the PI3K/Akt and ERK1/2 pathways. Our results reveal a potential drug target for treating myocardial I/R injuries.

## Materials and methods

### Reagents and antibodies

DMEM and foetal bovine serum (FBS) were purchased from Invitrogen (Carlsbad, CA, USA). Recombinant human bFGF was purchased from Sigma-Aldrich (St. Louis, MO, USA). Anti-Akt, p-Akt (Ser473), anti-ERK1/2, p-ERK1/2 (Thr202/Tyr204), anti-cleaved-caspase-3, cleaved-caspase-9, Bax, Bcl-2, cleaved-PARP, cytochrome *c*, anti-CHOP, cleaved-caspase-12, glucose-regulated protein (GRP-78), ATF-6 and GAPDH antibodies were purchased from Santa Cruz Biotechnology (Santa Cruz, CA, USA). Goat antirabbit and antimouse IgG-HRP were purchased from Cell Signaling Technology, Inc. (Danvers, MA, USA). An enhanced chemiluminescence (ECL) kit was purchased from Bio-Rad (Hercules, CA, USA). TBHP, PI3K/Akt inhibitor LY294002, ERK1/2 inhibitor PD98059 and all other reagents were purchased from Sigma-Aldrich unless otherwise specified.

### Cell culture and viability assay

Rat cardiomyocyte H9C2 cells were purchased from the American Type Culture Collection and maintained in DMEM containing 10% foetal bovine serum under 5% CO_2_. H9C2 cells were seeded on 96-well plates and treated with different doses of the TBHP for 8 hrs. To determine the effective concentration for cytoprotection, cells were also pre-treated with various concentrations of recombinant bFGF. Cell viability was assessed using the methyl thiazolyl tetrazolium assay. To further evaluate the effect of PI3K/Akt and ERK1/2 activation on oxidative injury, cells were pre-treated for 2 hrs with specific inhibitors, namely LY294002 (20 μM) and PD98059 (20 μM), before the addition of TBHP, as previously described. All experiments were performed in triplicate.

### Myocardial I/R model in mice and bFGF treatment

Adult male C57/B6 mice (8–12 weeks of age) were supplied by the Animal Center of the Chinese Academy of Sciences. The animal use and care protocol conformed to the Guide for the Care and Use of Laboratory Animals from the National Institutes of Health and was approved by the Animal Care and Use Committee of Jilin University. Experimental MI/R was induced by transient myocardial ischaemia for 30 min. and was followed by reperfusion for 4 hrs, as described previously. Mice were anaesthetized with 4% chloral hydrate (100 mg/kg, i.p) and placed on a ventilator (Harvard Rodent Ventilator, Harvard Apparatus, Holliston, MA, USA) in the right lateral decubitus position, and core temperature was maintained at 37°C with a heating pad. After a left lateral thoracotomy and pericardiectomy, the left anterior descending coronary artery was occluded for 30 min. with an 8-0 nylon suture and polyethylene tubing to prevent arterial injury and then re-perfused for 4 hrs. The mice that survived surgery were assigned randomly to the different treatment groups (*n* + 6–15 per group). Control group operations were performed in which animals underwent the same surgical procedure without coronary artery ligation (*n* + 4–8 per group). The I/R mice were administered 2 μg bFGF/mouse through intramyocardial injection at 30 min. after ischaemia.

### TUNEL assay

DNA fragmentation *in vivo* was detected using a one-step TUNEL Apoptosis Assay KIT (Roche, Mannheim, Germany). The images were captured with a Nikon ECLIPSE Ti microscope (Nikon, Melville, NY, USA). The apoptotic rates of the H9C2 cells treated with TBHP and bFGF were measured using a PI/Annexin V-FITC kit (Invitrogen) and then analysed by a FACScan flow cytometer (Becton Dickinson, Franklin Lakes, NJ, USA) according to the kit's manual.

### Fluorescence activated cell sorting (FACS) analysis

The cells were cultured at a density of 2 × 10^5^ cells per well in growth medium for 24 hrs in 6-well plates. The cells were then pre-incubated with 50 nM bFGF which was followed 2 hrs later by exposure to 100 μM TBHP for 8 hrs. Meanwhile, inhibitors of PI3K and ERK phosphorylation were added to the cells 2 hrs prior to TBHP at a final concentration of 20 μM. Annexin V assays were performed with the Annexin V-FITC Apoptosis Detection Kit (Becton Dickinson, San Jose, CA, USA). Cells were washed twice with cold PBS and re-suspended in binding buffer before the addition of Annexin V-FITC and propidium iodide (PI). Cells were vortexed and incubated for 15 min. in the dark at room temperature before analysis using a FACSCalibur flow cytometer (BD Biosciences, San Jose, CA, USA) and FlowJo software (Tree Star, San Carlos, CA, USA).

### Immunofluorescence Staining

To determine CHOP, GRP-78, cleaved-PARP and cleaved caspase-12 activities, sections were incubated with 0.3% H_2_O_2_ in methanol for 30 min., followed by blocking with 1% bovine albumin in PBS for 1 hr at room temperature. Next, the sections were incubated at 4°C overnight with a primary antibody against CHOP (1:200), GRP-78 (1:200), cleaved-PARP (1:200) or cleaved caspase-12 (1:1000). After primary antibody incubation, the sections were washed for 4 × 10 min. at room temperature and then incubated with donkey antimouse/rabbit, donkey antirabbit/mouse or donkey antigoat secondary antibody (1:500; Invitrogen) for 1 hr at room temperature. The saline injection group was considered the negative control. The images were captured using a Nikon ECLPSE 80i.

### Western blot

Total proteins were purified using protein extraction reagents for the heart tissue and H9C2 cells. The equivalent of 50 μg of protein was separated by 12% gel and then transferred onto a PVDF membrane. After blocking with 5% fat-free milk, the membranes were incubated with the relevant protein antibodies overnight. The membranes were washed with TBS and treated with secondary antibodies for 2 hrs at room temperature. The signals were visualized with the ChemiDicTM XRS + Imaging System (Bio-Rad Laboratories), and the band densities were quantified with Multi Gauge Software of Science Lab 2006 (FUJIFILM Corporation, Tokyo, Japan).

### Statistical analysis

Data are expressed as the mean ± SEM. Statistical significance was determined using Student's *t*-test when there were two experimental groups. When more than two groups were compared, statistical evaluation of the data was performed with one-way anova and Dunnett's *post hoc* test. *P* < 0.05 was considered statistically significant.

## Results

### bFGF decreases myocardial apoptosis in myocardial I/R mouse

**Fig 1:**
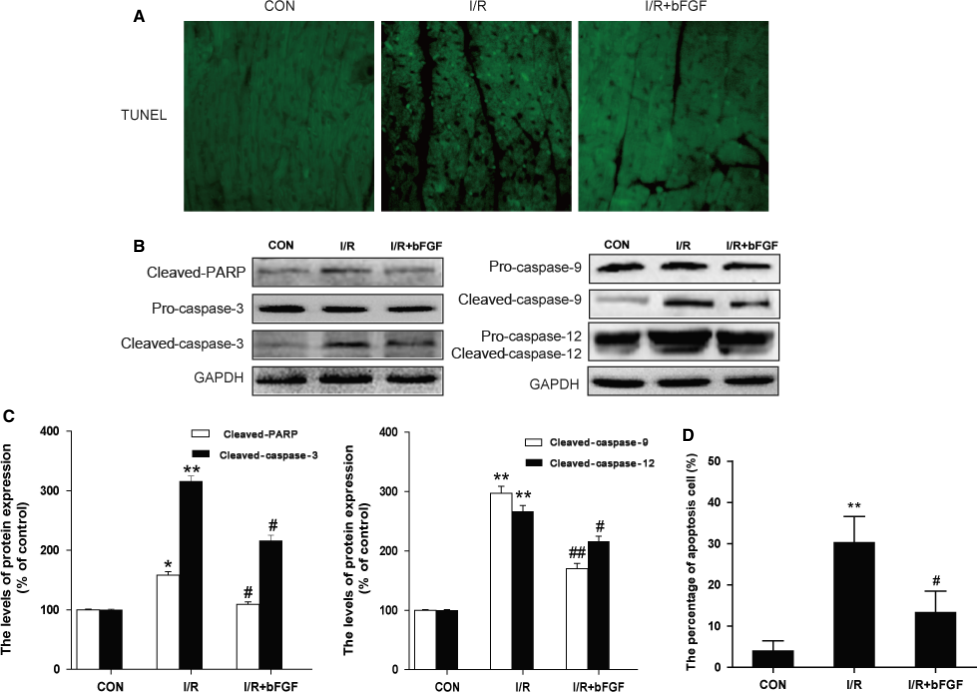
Basic fibroblast growth factor (bFGF) reduces myocardial apoptosis and the caspase cascade pathway in the hearts of mice after myocardial ischaemia/reperfusion. (A) Representative terminal deoxynucleotidyl transferase-mediated dUTP nick end labelling (TUNEL) immunofluorescence of sections from the ischaemic area in the hearts of mice that received bFGF or vehicle. (B) The detection of endoplasmic reticulum (ER) stress-related and mitochondrial dysfunction-related apoptosis proteins was performed by western blotting. The protein expression levels of cleaved-PARP, caspase-3, caspase-9 and caspase-12 in the hearts of control, ischaemia/reperfusion (I/R) mice and I/R mice treated with bFGF. (C) The optical density analysis of cleaved-PARP, caspase-3, caspase-9 and caspase-12 in the heart. (D) The percentage of apoptosis was counted from three random 1 mm^2^ areas. *P < 0.05, **P < 0.01, *versus* the Control group, ^#^*P* < 0.05, ^##^*P* < 0.01 *versus* the I/R group; *n* + 6.

To determine the role of bFGF in cardiac protection, bFGF was delivered into the mouse myocardium at 30 min. after ischaemia. The myocardial apoptosis in the myocardial I/R group was detected using TUNEL staining. As shown in Figure1A, there were no apoptosis-positive cells in the control group. The numbers of TUNEL-positive cells increased significantly after 4 hrs of ischaemia reperfusion, and the bFGF treatment group showed significant protective effects.

To further confirm the protective effect of bFGF, the protein expression levels of the caspase cascade pathway in the heart after myocardial ischaemia reperfusion were detected by western blot. We found that the levels of the cleaved-PARP, cleaved-caspase-3, cleaved-caspase-9 and cleaved-caspase-12 proteins decreased significantly after bFGF treatment compared with the I/R group 4 hrs after injury (Fig.1B and C). Our data indicated that bFGF administration has a cardioprotective effect and significantly reduces caspase cascade pathway activation.

### bFGF inhibits ER stress and mitochondrial dysfunction in myocardial I/R mouse

**Fig 2:**
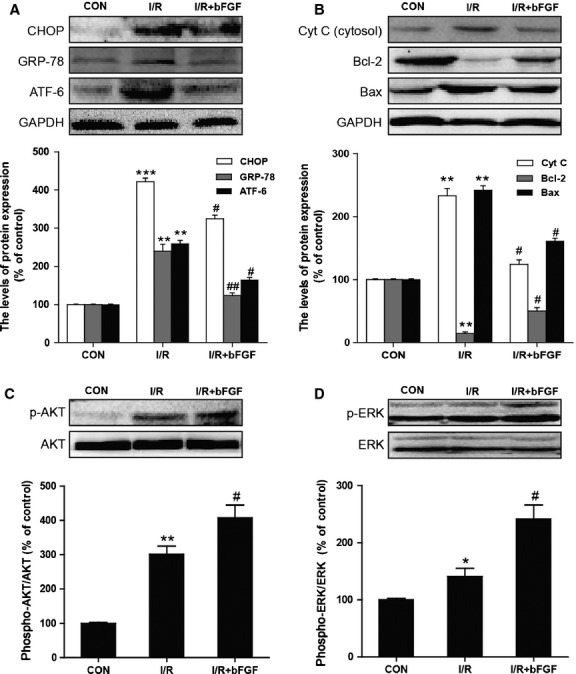
The effect of basic fibroblast growth factor (bFGF) on endoplasmic reticulum (ER) stress and mitochondrial dysfunction-related proteins in the hearts of mice after myocardial ischaemia/reperfusion (I/R). (A) The protein expression levels and optical density analysis of CHOP, GRP-78 and ATF-6 in the hearts of control, I/R mice and I/R mice treated with bFGF. (B) The protein expression levels and optical density analysis of Cyt c, Bcl-2 and Bax in the hearts of control, I/R mice and I/R mice treated with bFGF. (C) The protein expression levels and optical density analysis of p-AKT and AKT in the heart. (D) The protein expression levels and optical density analysis of p-ERK and ERK in the heart. *P < 0.05, **P < 0.01, *versus* the control group, ^*#*^*P* < 0.05, ^*##*^*P* < 0.01 *versus* the I/R group; *n* + 6.

**Fig 3:**
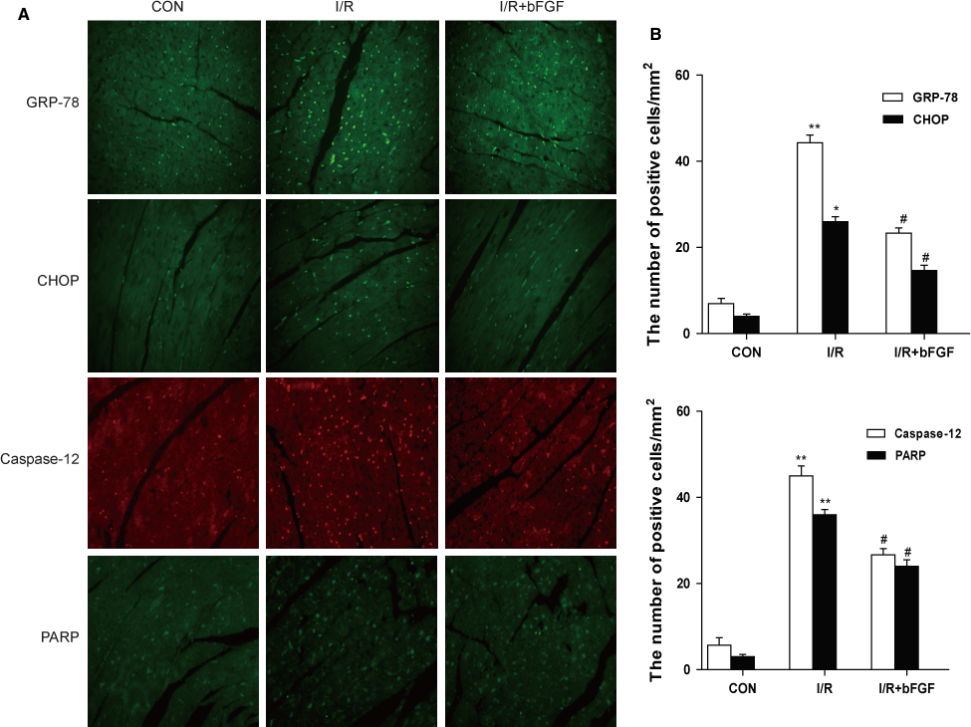
Immunofluorescent staining of endoplasmic reticulum (ER) stress and mitochondrial dysfunction-related proteins in the hearts of mice. (A) Immunofluorescent staining for GRP-78, CHOP, cleaved caspase-12 and cleaved-PARP in the hearts of control, ischaemia/reperfusion (I/R) mice and I/R mice treated with basic fibroblast growth factor (bFGF). (B) Analysis of the positive cells of the immunofluorescent results. *P < 0.05, **P < 0.01, *versus* the control group, ^*#*^*P* < 0.05, *versus* the I/R group; *n* + 5.

To determine whether the cardioprotective effect of bFGF is related to ER stress and mitochondrial dysfunction, we measured the expression of ER stress and mitochondrial dysfunction proteins. Our western blot results indicated that the protein levels of C/EBP homologous protein (CHOP), 78 kD GRP-78, ATF-6 and cleaved caspase-12 were significantly up-regulated in the hearts of I/R mice when compared with the sham group. Moreover, bFGF treatment inhibited the activation of ER stress-related proteins in the hearts of I/R mice (Fig.2A). We also determined *via* CHOP, GRP-78 and cleaved caspase-12 immunofluorescent analysis that there are few ER stress protein-positive cells in the control group. The numbers of ER stress protein-positive cells increased significantly after 4 hrs of ischaemia reperfusion, and the bFGF treatment group showed significant protective effects (Fig.3A). In addition, western blot and immunofluorescent results all suggested that bFGF inhibits the up-regulation of mitochondrial dysfunction-related proteins cytochrome c (Cyt c), Bax and Bcl-2, which were induced by I/R injury (Figs2B and 3A). To further understand the mechanism underlying behind the effect of bFGF on I/R injury, the activation of PI3K/Akt and ERK1/2 downstream signals were also analysed by western blot. As expected, bFGF treatment increased the phosphorylation of Akt and ERK1/2 in the hearts of I/R mice when compared with controls (Fig.2C and D). Taken together, these results suggest that the protective role of bFGF in I/R injury is related to the inhibition of ER stress and mitochondrial dysfunction through the activation of the PI3K/Akt and ERK1/2 signalling pathways.

### bFGF reduces oxidative stress-induced cell death in H9C2 cells

**Fig 4:**
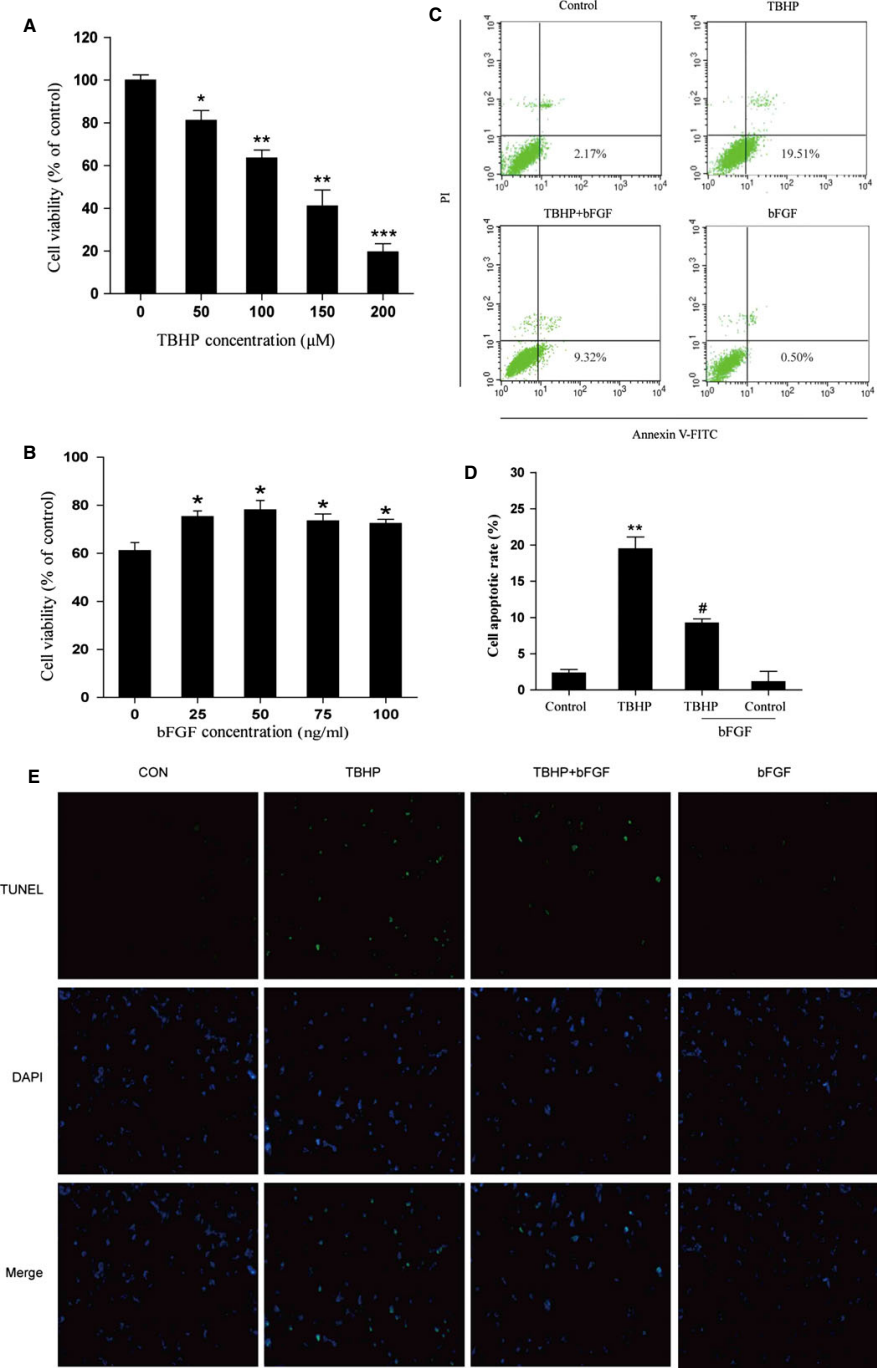
Basic fibroblast growth factor (bFGF) inhibits apoptosis induced by hydroperoxide (TBHP) in H9C2 cells. (A) PC12 cells were treated with different concentrations of TBHP (0, 50, 100, 150, 200 μM) for 8 hrs, and then cell viability was assessed by the 3-(4,5-Dimethylthiazol-2-yl)-2,5-diphenyltetrazolium bromide (MTT) assay. (B) H9C2 cells were pre-treated with bFGF (0, 25, 50, 75, 100 ng/ml) for 2 hrs, 100 μM TBHP was added for an additional 8 hrs, and then cell viability was assessed by the MTT assay. (C) H9C2 cells were pre-treated with 50 ng/ml bFGF for 2 hrs, and then 100 μM TBHP was added for an additional 8 hrs. Cells were then stained with Annexin V-FITC/propidium iodide and detected by flow cytometry; the lower right panel indicates the apoptotic cells. (D) Bar diagram of apoptotic cell rates from three separate experiments. (E) Detection of apoptotic cells by TUNEL (green) and DAPI (blue) staining assay. *P < 0.05, **P < 0.01, *versus* the control group, ^#^*P* < 0.05, ^##^*P* < 0.01 *versus* the TBHP group.

To establish a suitable concentration of TBHP and bFGF in H9C2 cells, we conducted dose–response experiments (Fig.4A) and determined that TBHP incubation resulted in dose-dependent cell death after 8 hrs exposure. In addition, bFGF increased cell viability at concentrations under 75 ng/ml (Fig.4B). Based on these data, 100 μM TBHP and 50 ng/ml bFGF were chosen for subsequent experiments. To further confirm the effect of bFGF on TBHP-induced apoptosis, cells were subjected to PI and Annexin V-FITC staining, and the apoptotic cells were then quantified by FACS. As shown in Figure4C and D, TBHP-induced apoptosis was decreased after treatment with bFGF. The apoptotic cells were also detected by TUNEL assay, producing results consistent with the flow cytometry results indicating that apoptosis was suppressed by bFGF treatment. Our results suggested that bFGF inhibits TBHP-induced apoptosis and may play a protective role in oxidative stress injury in H9C2 cells.

### bFGF decreases ER stress and mitochondrial dysfunction-induced apoptosis in H9C2 cells

**Fig 5:**
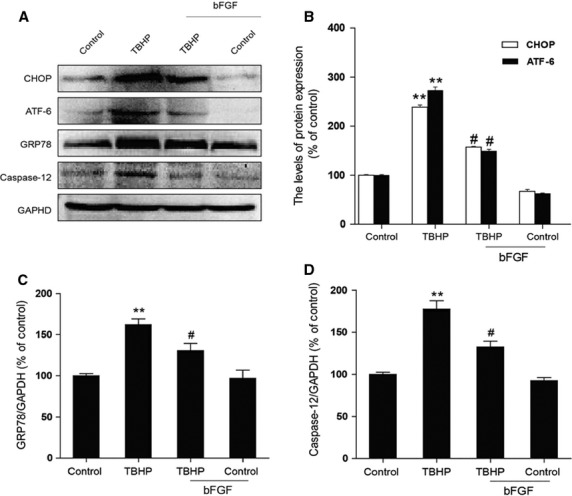
Basic fibroblast growth factor (bFGF) attenuates endoplasmic reticulum (ER) stress-related proteins induced by hydroperoxide (TBHP) in H9C2 cells. (A) H9C2 cells were pre-treated with 50 ng/ml bFGF for 2 hrs, and then 100 μM TBHP was added for an additional 8 hrs. The cell lysates were analysed for the expression of CHOP, GRP-78, ATF-6 and caspase-12 by western blotting. Bar diagram of (B) CHOP, ATF-6, (C) GRP-78 and (D) caspase-12 expression from three Western blot analyses. *P < 0.05, **P < 0.01, *versus* the control group, ^#^*P* < 0.05, ^##^*P* < 0.01 *versus* the TBHP group.

**Fig 6:**
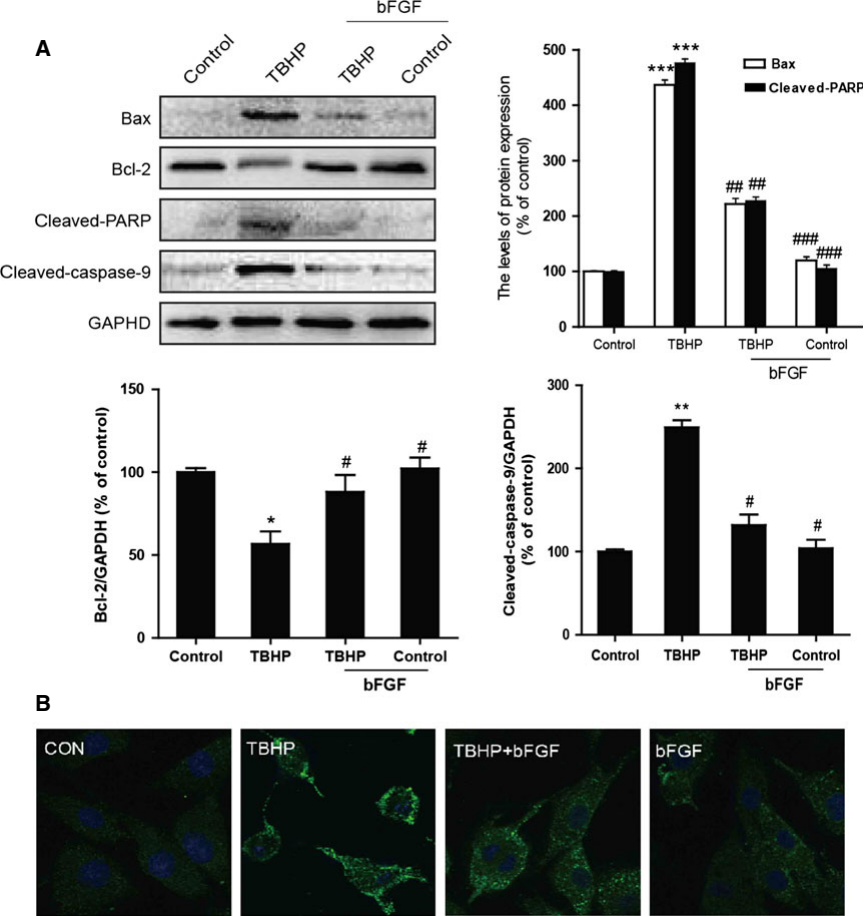
The effect of basic fibroblast growth factor (bFGF) on mitochondrial dysfunction-related proteins induced by hydroperoxide (TBHP) in H9C2 cells. (A) H9C2 cells were pre-treated with 50 ng/ml bFGF for 2 hrs, and then 100 μM TBHP was added for an additional 8 hrs. The cell lysates were analysed for the expression of Bax, Bcl-2, cleaved-PARP and cleaved-caspase-9 by western blotting. Bar diagram of Bax, Bcl-2, cleaved-PARP and cleaved-caspase-9 expression from three Western blot analyses. (B) Immunofluorescence results of the mitochondrial apoptotic marker cytochrome c in H9C2 cells. *P < 0.05, **P < 0.01, *versus* the control group, ^#^*P* < 0.05, ^##^*P* < 0.01 *versus* the TBHP group.

To investigate whether the apoptosis induced by TBHP and the effect of bFGF were related to chronic ER stress and mitochondrial dysfunction in H9C2 cells, the levels of the ER stress and mitochondrial dysfunction-related proteins were measured by western blot or immunofluorescent analysis. As shown in Figure5, the expression of CHOP, cleaved caspase-12, GRP-78 and ATF-6 were higher in the TBHP-treated group than in the control group. bFGF treatment significantly inhibited the ER stress-related proteins that had been induced by TBHP. On the other hand (Fig.6A and B), the mitochondrial dysfunction-related proteins Bax, Bcl-2, cleaved-PARP, cleaved-caspase-9 and cytochrome *c* were also significantly increased in the TBHP-treated group, whereas these proteins attenuated significantly after bFGF treatment. Taken together, our data suggest that the protective role of bFGF may involve the inhibition of ER stress-related and mitochondrial dysfunction-related proteins.

### bFGF activate the PI3K/Akt and ERK1/2 pathways

**Fig 7:**
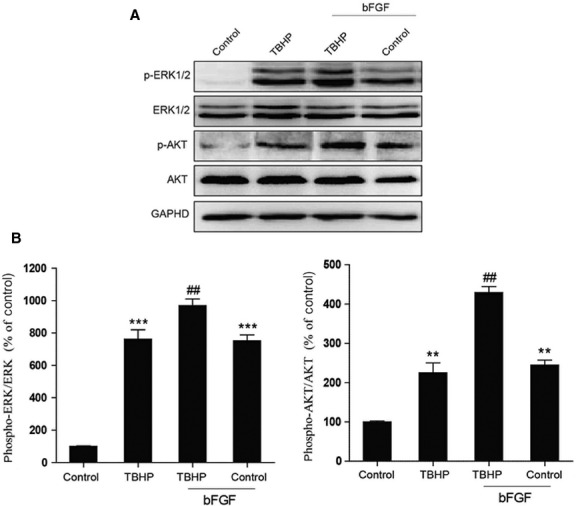
Basic fibroblast growth factor (bFGF) activates PI3K/Akt and ERK1/2 in H9C2 cells. (A) H9C2 cells were pre-treated with 50 ng/ml bFGF for 2 hrs, and then 100 μM hydroperoxide (TBHP) was added for an additional 8 hrs. The cell lysates were analysed by western blotting for the expression of phospho-Akt, Akt, phospho-ERK and ERK. (B) Bar diagram of phospho-Akt/Akt and phosphorylated-ERK/ERK levels from three Western blot analyses. GAPDH was used as a protein loading control and for band density normalization. *P < 0.05, **P < 0.01, *versus* the control group, ^#^*P* < 0.05, ^##^*P* < 0.01 *versus* the TBHP group.

Based on our previous experiments, we hypothesized that the PI3K/Akt and ERK1/2 pathways may be involved in the bFGF-mediated inhibition of ER stress and mitochondrial dysfunction in an oxidative stress injury model. As shown in Figure7A and B, an increase in p-Akt and p-ERK1/2 was observed in the cells exposed to TBHP compared with the control group. The pre-treatment of bFGF significantly increased the activation of the PI3K/Akt and ERK1/2 pathways in the H9C2 cells exposed to TBHP. These data suggest that both the PI3K/Akt and ERK1/2 pathways are involved in the protective effect of bFGF.

### Inhibition of the PI3K/Akt and ERK1/2 pathway partially reverses the protective effect of bFGF

**Fig 8:**
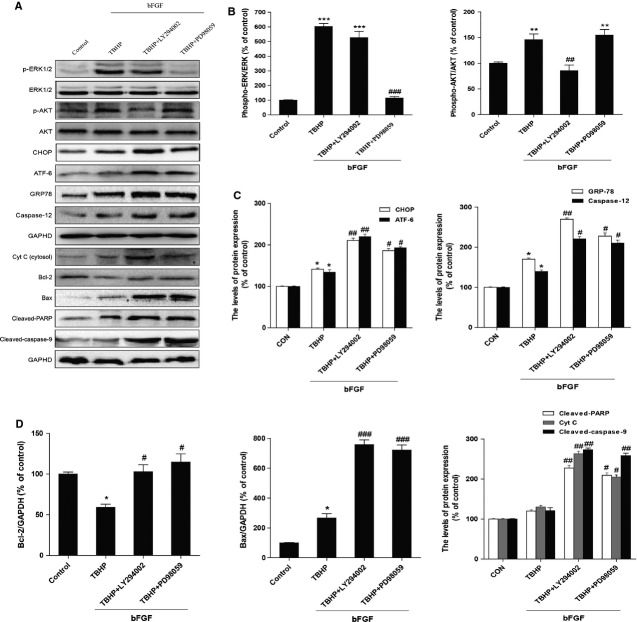
Inhibition of the PI3K/Akt and ERK1/2 pathways partially attenuates the basic fibroblast growth factor (bFGF)-mediated reduction in the endoplasmic reticulum (ER) stress and mitochondrial dysfunction effects in H9C2 cells. H9C2 cells were pre-treated with 50 ng/ml bFGF with or without the specific inhibitors LY294002 (20 μM) and PD98059 (20 μM) for 2 hrs, and then 100 μM hydroperoxide (TBHP) was added for an additional 8 hrs. The cell lysates were analysed by western blotting to detect the expression of phospho-Akt, phospho-ERK and ERK, CHOP, GRP-78, ATF-6, caspase-12, Bax, Bcl-2, Cyt c, cleaved-PARP and cleaved-caspase-9. Bar diagram of the (B) phospho-Akt/Akt ratio, phospho-ERK/ERK ratio, (C) CHOP, GRP-78, ATF-6 and caspase-12, (D) Bax, Bcl-2, Cyt c, cleaved-PARP and cleaved-caspase-9 expression from three Western blot analyses. GAPDH was used as a protein loading control and for band density normalization. *P < 0.05, **P < 0.01 and ***P <* *0.001 *versus* the control group, ^#^*P* < 0.05, ^##^*P* < 0.01 *versus* the TBHP group.

**Fig 9:**
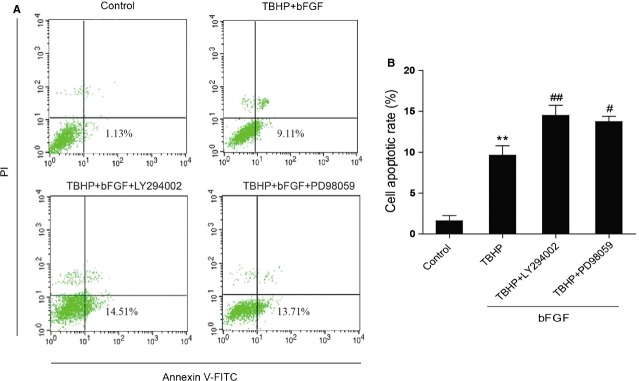
Inhibition of PI3K/Akt and ERK1/2 pathways partially impairs the protective effect of basic fibroblast growth factor (bFGF) in H9C2 cells. (A) H9C2 cells were pre-treated with 50 ng/ml bFGF with or without the specific inhibitors LY294002 (20 μM) and PD98059 (20 μM) for 2 hrs, and then 100 μM hydroperoxide (TBHP) was added for an additional 8 hrs. Cells were collected and stained with Annexin V-FITC/propidium iodide and detected by flow cytometry; the lower right panel indicates the apoptotic cells. (B) Bar diagram of the apoptotic cell rate from three separate experiments. *P < 0.05, **P < 0.01 *versus* the control group, ^#^*P* < 0.05, ^##^*P* < 0.01 *versus* the TBHP group.

To further confirm the important role of PI3K/Akt and ERK1/2 activation by bFGF, the PI3K inhibitor LY294002 and the ERK1/2 inhibitor PD98059 were added to the media. LY294002 and PD98059 inhibited the activation of Akt and ERK1/2, respectively, when compared with the bFGF group (Fig.8A and B). Moreover, the expression of CHOP, GRP-78, cleaved caspase-12 and ATF-6 increased after the combined exposure to LY294002 and PD98059 when compared with the bFGF treatment group (Fig.7A and C). In addition, LY294002 and PD98059 can also reverse the inhibitory effect of bFGF on the expression of Bax, Bcl-2, cleaved-PARP, cleaved-caspase-9 and Cyt c. These results suggest that the mitochondrial dysfunction and ER stress-induced apoptosis were aggravated by the addition of Akt or ERK inhibitors. The apoptotic cell rate was determined by FACS. As shown in Figure9A and B, the addition of LY294002 or PD98059 significantly increased cell apoptosis when compared with the bFGF group. All of these results suggest that the protective effect of bFGF is mediated by both the PI3K/Akt and ERK1/2 signalling pathways.

## Discussion

Ischaemic heart disease secondary to acute myocardial infarction, is among the most prevalent health problems in the world and is a major cause of morbidity and mortality. After ischaemia, myocardial reperfusion is followed by a long period of secondary myocardial injury that may include oxidative stress, inflammation, necrosis and apoptosis. The loss of myocardial cells is the main factor that interferes with recovery from the secondary damage. Several studies have shown that bFGF delivered during reperfusion protects against ischaemia-reperfusion injury 25,26. However, as a multifunctional factor, the protective effect of bFGF on oxidative stress-induced cell death observed in the myocardial ischaemia/reperfusion model is not yet fully understood. In our previous study, we confirmed that the administration of bFGF immediately after reperfusion significantly protected the heart from I/R-induced injuries 7. However, the molecular mechanism of bFGF in the treatment of myocardial injury in the early-phase after reperfusion is still unclear. Therefore, in this study, we aimed to define the molecular mechanism of bFGF treatment in recovery from myocardial I/R injuries. Specifically, we examined the therapeutic effect of bFGF on ER stress-induced and mitochondrial dysfunction-induced apoptosis both *in vitro* and *in vivo*.

Moreover, several studies have indicated that elevated levels of ROS in myocardial I/R injuries increase calcium release from internal stores. Two cytoplasmic organelles are involved in Ca^2+^ homoeostasis - ER and mitochondria. Changes in mitochondrial membrane permeability induced by ROS lead to the leakage of Ca^2+^ from the mitochondrial matrix into the cytosol and elevate intracellular levels of Ca^2+^ 27. A number of cellular stress conditions including the depletion of intracellular Ca^2+^ stores and oxidative stress lead to the accumulation of unfolded or misfolded proteins in the ER lumen, which is referred to as ‘ER stress’ 28.

Oxidative stress-induced mitochondrial dysfunction is an important gate-keeper of life and death in the cardiomyocyte. In cardiac myocytes, the mitochondria occupy up to 30% of the total volume and provide energy to the contracting cell in the form of ATP *via* oxidative phosphorylation. The mitochondria are also very sensitive to alterations in the cellular environment and can quickly switch from being supporters of life to promoters of cell death. Therefore, it is not surprising that mitochondrial dysfunction is associated with the loss of myocytes and the subsequent development of heart failure. It is well known that myocardial ischaemia and reperfusion results in mitochondrial dysfunction and cell death *via* necrosis and apoptosis. Apoptosis is activated by the permeabilization of the outer mitochondrial membrane and the release of pro-death proteins, such as cytochrome c, by the pro-apoptotic Bcl-2 members Bax and Bak 15. The Bcl-2 proteins have been found to play major roles in I/R. For instance, transgenic mice overexpressing Bcl-2 in the heart had reduced levels of apoptosis, smaller infarcts and improved cardiac function after I/R compared with wild-type mice 29,30. Similarly, Bax-deficient mice had reduced mitochondrial damage and infarct size after I/R 31. In this study, the levels of these mitochondria-induced apoptosis proteins after myocardial I/R injuries were detected *in vitro* and *in vivo* to investigate the molecular mechanism of bFGF during recovery from I/R injury. We found that the expression levels of Bcl-2, Bax, cytosolic Cyt *c*, cleaved-PARP and cleaved-caspase-9 increased significantly after I/R injury. Exogenous bFGF treatment after I/R decreased the levels of mitochondria-related proteins and inhibited apoptosis. These results indicated that the protective role of bFGF in myocardial I/R injury is related to the inhibition of mitochondrial response proteins.

There is increasing evidence that oxidative stress-induced ER stress plays a crucial role in I/R-induced cell dysfunction and death. In the cell culture model of oxygen and glucose deprivation, the ER stress response protein CHOP was up-regulated and found to induce cardiomyocyte death. The ER stress pathway was first identified as a cellular response induced by the accumulation of unfolded proteins in the ER to preserve the organelle's function. The ER stress pathway is also activated by various cellular stress processes that may induce apoptosis 32. Previous studies have demonstrated that ER stress is one of the main events in the pathogenesis of myocardial I/R injury 33,34. Pharmacological inhibition of ER stress could prevent infarct size reduction because of the inhibition of CypD in the mouse model of acute myocardial infarction 35. The ER, also called the sarcoplasmic reticulum in cardiomyocytes, is responsible for synthesizing, modifying, folding and trafficking secretory proteins as well as regulating intracellular Ca^2+^ homoeostasis. It is highly sensitive to the deprivation of cellular energy and alterations of the redox state or Ca^2+^ concentration. These changes lead to the accumulation of unfolded proteins - a condition called ‘ER stress’ 36. In response to ER stress, GRP78 an ER chaperone, is significantly up-regulated. The GRP78 level is normally regarded as an indicator of ER stress, and a previous report demonstrated a significant up-regulation of GRP78 levels following 12 hrs ischaemia and 4 hrs reperfusion 37. Excessive and prolonged ER stress can trigger cell death, typically apoptosis. CHOP and caspase-12 are two specific mediators of ER stress-induced apoptosis pathways 38. We found that the expression levels of GRP78, CHOP, ATF-6 and caspase-12 increased significantly after I/R injury. Exogenous bFGF treatment after I/R decreased the levels of ER stress-related proteins and inhibited apoptosis. These results indicate that the protective role of bFGF in myocardial I/R injury is related to the inhibition of ER stress response proteins.

The PI3K/Akt and ERK1/2 pathways are two main downstream signals activated by bFGF to produce the cardioprotective effects. The PI3K/Akt pathway is particularly important for mediating myocardial survival under a wide variety of circumstances 39,40. Akt functions before the release of Cyt c by regulating Bcl-2 family member activity and mitochondrial function as well as components of the apoptosome 41,42. For instance, Akt phosphorylates the pro-apoptotic Bcl-2 family member Bax and thereby inhibits Bax's pro-apoptotic functions 43. In myocardial I/R injuries, it has been reported that PI3K/Akt partially mediates Danshensu-mediated survival. Another study revealed that cardioprotection by Danshensu is also partially depends partially upon ERK1/2 signalling 44. In our previous study, bFGF shows a neuronal protective effect in a stroke rat model *via* the activation of both the PI3K/Akt and ERK1/2 signals 6. We also proved that bFGF prevents SCI-induced apoptosis in spinal cord contusions by activating both the PI3K/Akt and ERK1/2 pathways 45. In this study, we demonstrated that the role of bFGF in myocardial I/R injury recovery is also related to the activation of both the PI3K/Akt and ERK1/2 pathways. To further confirm that these two pathways are essential for the protective effect of bFGF, we used the PI3K/Akt inhibitor LY294002 or the ERK1/2 inhibitor PD98059 to treat H9C2 cells and show that the apoptosis induced by TBHP was inhibited by bFGF treatment and further abolished by this combination of inhibitors.

However, it is no doubt that the limitations of bFGF in myocardial I/R injury therapy still need further investigations and improvements. For example, bFGF is not stable enough which is easy to be degraded by various enzymes *in vitro*, resulting in the loss of biological activity. So the combination with other drugs or delivery systems to increase its stability may contribute to the functions of bFGF. Moreover, H9C2 cell used *in vitro* is informative, future tests in primary culture cardiac myocyte would be more translational which will indicates the role of bFGF in the cardiac myocyte death directly. Nevertheless, the protective effect of bFGF in the therapy of myocardial I/R injury is confirmative and feasible, to explore the appropriate therapeutic time window and special drug delivery system combined with factors still need long-term study.

In conclusion, bFGF treatment significantly reduced the apoptosis induced by acute myocardial I/R injuries, which is related to the inhibition of ER stress- and mitochondrial-related protein expression *via* both the PI3K/Akt and ERK1/2 signalling pathways. Our study demonstrates the possibility that bFGF therapy may be suitable for recovery from ischaemia/reperfusion heart disease.
